# α4-α5 Helices on Surface of KRAS Can Accommodate Small Compounds That Increase KRAS Signaling While Inducing CRC Cell Death

**DOI:** 10.3390/ijms24010748

**Published:** 2023-01-01

**Authors:** Baraa Abuasaker, Eduardo Garrido, Marta Vilaplana, Jesús Daniel Gómez-Zepeda, Sonia Brun, Marta Garcia-Cajide, Caroline Mauvezin, Montserrat Jaumot, Maria Dolors Pujol, Jaime Rubio-Martínez, Neus Agell

**Affiliations:** 1 Departament de Biomedicina, Facultat de Medicina i Ciències de la Salut, Universitat de Barcelona, 08036 Barcelona, Spain; 2 Departament de Ciència de Materials i Química Física, Facultat de Química, Universitat de Barcelona & Institut de Recerca en Química Teòrica i Computacional (IQTCUB), 08028 Barcelona, Spain; 3 Departament de Farmacologia, Toxicologia i Química Terapèutica, Facultat de Farmàcia i Ciències de l’Alimentació, Universitat de Barcelona, 08028 Barcelona, Spain

**Keywords:** colorectal cancer, KRAS, molecular dynamics, docking, allosteric pocket, calmodulin, small molecule inhibitors, RAF, ERK, AKT

## Abstract

KRAS is the most frequently mutated oncogene associated with the genesis and progress of pancreatic, lung and colorectal (CRC) tumors. KRAS has always been considered as a therapeutic target in cancer but until now only two compounds that inhibit one specific KRAS mutation have been approved for clinical use. In this work, by molecular dynamics and a docking process, we describe a new compound (P14B) that stably binds to a druggable pocket near the α4-α5 helices of the allosteric domain of KRAS. This region had previously been identified as the binding site for calmodulin (CaM). Using surface plasmon resonance and pulldown analyses, we prove that P14B binds directly to oncogenic KRAS thus competing with CaM. Interestingly, P14B favors oncogenic KRAS interaction with BRAF and phosphorylated C-RAF, and increases downstream Ras signaling in CRC cells expressing oncogenic KRAS. The viability of these cells, but not that of the normal cells, is impaired by P14B treatment. These data support the significance of the α4-α5 helices region of KRAS in the regulation of oncogenic KRAS signaling, and demonstrate that drugs interacting with this site may destine CRC cells to death by increasing oncogenic KRAS downstream signaling.

## 1. Introduction

KRAS was the first oncogene identified in human cancer [1] and activating mutations affecting members of the RAS family genes (KRAS, HRAS, NRAS) are the most frequent genetic alterations, being found in about 27% of all tumors. KRAS is the most recurrently mutated gene (86% of RAS-mutant cancers) [2,3] and is a driver gene that is particularly involved in the development and progression of pancreatic (PDAC), lung and colorectal (CRC) cancers (for recent reviews see [4,5]).

KRAS encodes for a small GTPase that in normal cells functions as a molecular switch cycling from a GDP-bound inactive to a GTP-bound active state [6,7]. In quiescent cells, KRAS is bound to GDP and, upon activation of guanidine exchange factors (GEFs) in response to proliferative external signals, the GDP is released and GTP binds immediately to KRAS. In the GTP-bound form KRAS can interact with its effectors which in turn initiate specific signal transduction pathways, inducing the cell response. The main KRAS effectors are RAF, PI3K and Ral-GDS. Binding of RAF to KRAS induces unlocking of the closed conformation of RAF favoring its activation and consequently MEK and ERK activation. PI3K is also stimulated upon binding to KRAS leading to AKT activation. Appropriate activation of ERK and AKT will lead to cell proliferation and survival. Interestingly, other proteins able to interact with GTP-bound KRAS are GTPase activating proteins (GAPs), which, by stimulating the endogenous GTPase activity of KRAS, induce the transition to GDP-bound KRAS and consequently the shutdown of the downstream signaling. Most common oncogenic mutations of KRAS (G12 mutations, 83% of all KRAS alterations) [8,9] produce proteins with null GTPase activity, thus becoming almost blocked in their GTP-bound state and consequently constitutively activating the downstream signaling pathways. This prompts cells to enter the cell cycle independently of the external proliferative signals—one of the hallmarks of cancer—although it is important to point out that overly sustained activation of ERK signaling can also have other physiological outputs such as differentiation or apoptosis. Hence, cells have diverse negative feedbacks to downregulate this pathway [4,10,11].

Targeting KRAS in cancer has been a central goal during the past four decades, but it is not until recently that all these efforts have started to bear fruit [5,12]. Sotorasib (AMG 510) and Adagrasib (MRTX849) are compounds that covalently bind to and block the oncogenic KRASG12C mutant and have been approved as second-line treatment for non-small cell lung cancer patients (NSCLC) with the KRASG12C-positive mutation [13,14]. Unfortunately, the frequency of this specific mutation is very low in PDAC and CRC and therefore other inhibitors need to be developed [3].

Increasing knowledge of the KRAS structure thanks to crystallography (reviewed in [9], NMR [15] and molecular dynamic (MD) modeling techniques [16] is allowing the development of new compounds with potential use in cancer therapy. KRAS consists of a G-domain (1-166) that is highly conserved in the other RAS family members (HRAS and NRAS) and a hypervariable (HVR) tail that is involved in membrane localization. The HVR of KRAS contains a polybasic domain and a cysteine that is farnesylated, which both favor the interaction of KRAS with the plasma membrane. The G-domain exists as two lobes. The first lobe (1-86 aa), which is identical in the three RAS isoforms, contains a region essential for effectors recognition (effector-binding domain: switch I, aa 30-38) and another crucial for GAPs and GEFs binding (switch II, aa 70-78), as well as the P-loop. The second lobe differs slightly between the different isoforms as it is the allosteric lobe. This lobe is important for establishing contact with the plasma membrane [17,18,19], for allowing KRAS dimerization [20,21,22,23], for interacting with the HVR [24], and for permitting interaction with other proteins such as calmodulin (CaM) (Garrido et al., 2018), the tumor suppressor DIRAS3 [25], and the cysteine-rich domain of RAF proteins [9,26]. Therefore, this lobe could be important for regulating the activity, not only of wild type KRAS, but also of oncogenic KRAS.

Knowledge of the structure of the switch I and II domains within the first lobe has allowed the design of molecules that break KRAS binding with its effectors, which may be potentially therapeutic [27,28,29,30,31,32]. In addition, different pockets and domains that can accommodate compounds have been defined in the allosteric lobe and, interestingly, some of these compounds can inhibit KRAS signaling and reduce cancer cells’ viability or tumor progression in mouse models [16,33,34,35]. Since we have previously shown that the α4-helix and the α5-helix on the surface of the allosteric domain of KRAS are important for the interaction of KRAS with CaM [36,37], and that CaM modulates KRAS activity [38,39,40], we further analyzed this region by MD for the presence of a putative druggable pocket and to identify compounds that stably bind KRAS. In this work, we describe a compound that interacts with KRAS and increases oncogenic KRAS signaling, but that remarkably reduces the viability of CRC cells expressing oncogenic KRAS.

## 2. Results

### 2.1. KRAS Modelling

**Figure 1:**
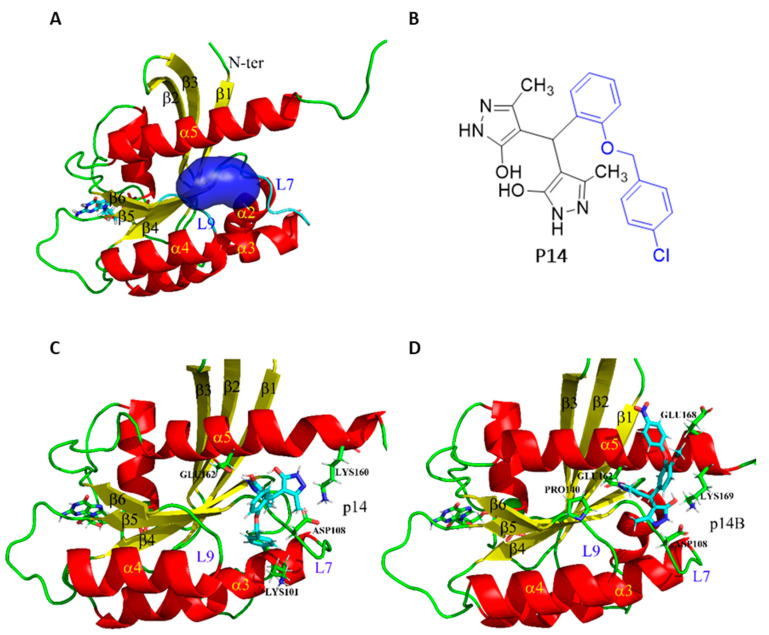
(**A**) KRAS binding site for calmodulin (CaM). Representation in blue surface of the binding site suggested both experimentally [37] and theoretically [36]. (**B**) Formula of the compound P14. (**C**,**D**) Spatial representation of the KRAS-P14 and KRAS-P14B complex at the end of 100ns of conventional molecular dynamics.

After determining the possible allosteric binding sites, as explained in the Materials and Methods Section, we analyzed the potential binding site near α helix 5 (Figure 1A) since this region was identified as the binding site for CaM both experimentally [37] and theoretically [36]. The existence of this pocket was further confirmed using LIGSITECSC [41] and Pocket-Depth [42]. Moreover, it is noted that this pocket is present in all the studied structures, even in the inactive state of KRAS. Consequently, the current study focused on the structure with code PDB id 4DSN, which corresponds to KRAS with the oncogenic mutation G12D and with the GTP cofactor loaded, thus turning it into the ideal target. Since the HVR region of KRAS possesses high motility, its structure has not yet been determined by X-ray crystallography. The missing residues (from 178 to 188) were added to the protein to obtain the full-length KRAS. To confirm that the joining of the HVR had not affected KRAS behavior, the HVR was attached in several other angles (adding 20, 70, 100 or 140 degrees relative to the initial position). Then, as explained in more detail in the Materials and Methods Section, the system was heated, the density stabilized and 50 ns of conventional molecular dynamics (cMD) were performed. No major differences were observed, as in all cases the HVR moved in random directions, interacting with the globular domain occasionally but without maintaining a significant interaction. These results suggested that the simulations were reliable and that the initial angle of attachment of the HVR was not relevant, as in 50 ns of dynamics the systems could sample similar regions. Afterwards, the length of the cMD for the initial system was extended up to 80 ns and the stability of the system was studied by performing an MMPB/GBSA analysis of the interaction between KRAS and GTP (Figure S1). From this cMD, five structures were selected for the docking process to provide pocket heterogeneity and conformational diversity in the KRAS receptor. To carry out this selection, the bonding status of GTP was considered by analyzing their binding energy during the time provided by the MMPBSA calculation (Figure S1). First, the average value of the binding energy along the trajectory was calculated. Then, the first structure was selected as that structure with binding energy similar to the mean and closest to the X-ray crystallography structure. The other four structures were selected as those with either higher or lower binding energy with respect to the average value (two of each).

### 2.2. Virtual Screening

To perform the docking process, two different compound sets were selected: the ZINC Lead-Like database and the MOE Lead-Like database. First, compounds from the databases were processed, using the MOE software [43], to have the right protonation state, optimized geometries optimized and a pool of conformation. Next, a multistep virtual screening procedure was performed on each of the five KRAS representatives selected. In the first step, a rigid docking was performed, and the best 1000 compounds of each subset were then selected. In the second step, for the selected complex structures, a refined docking was performed where both the lateral chains of the pocket and the compound could move without restrictions. After completing the dockings studies, the best compounds were selected for use in the initial experimental studies. The selection process was, in brief, as follows: the compounds were selected either based on their ability to bind to all the structures of KRAS or based on their binding energy, without considering how many structures had managed to bind. Then, the binding energy of the best 200, for each database, was more accurately calculated using an MMPB/GBSA analysis (more details can be found in the Materials and Methods Section). Nineteen compounds with the best energy were selected as good candidates for KRAS interactors.

Finally, based on its availability and solubility and after a preliminary study analyzing the effect on tumor cells’ viability, compound P14 (Figure 1B) was selected to biochemically study its interaction with KRAS, and to analyze putative effects on treated cancer cells’ signaling and viability. Some derivatives of P14 were also included in the investigation (P14A to P14D) (Figure S4, Methods S1). For compound P14 and the most promising of its derivatives (see Section 2.3), P14B (see Section 2.3), a cMD of 100ns length was performed to analyze their interaction with KRAS. Figures S2 and S3 show the evolution of their binding energies, using the MMPB/GBSA approach. We can see that, although there were changes in energetics, especially for P14B, both remained stable and bonded. Moreover, the binding modes at the end of the molecular dynamics are shown in Figure 1C,D for compounds P14 and P14B, respectively. Both compounds remained at the binding site described for the KRAS/CaM interaction although their binding mode differed. Compounds P14 and P14B both have the same bipyrazole system that is expected to bind to the target through polar interactions and it is the substituent in the alpha position of the pyrazoles that produced changes in their binding mode. Specifically, p14 presents only one hydrogen bond with Glu162 stabilizing one of the symmetrical rings, while p14B presents two hydrogen bonds, one with Glu162 and another with Asp108—a fact that could stabilize both symmetrical rings.

### 2.3. CRC Cells Treated with P14 and P14B Show Increased Downstream Ras Signaling

**Figure 2:**
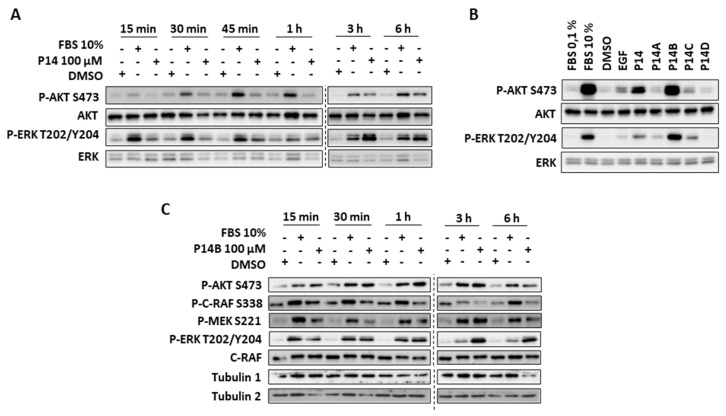
P14 and P14B treatment of DLD-1 cells increased endogenous downstream RAS signaling. (**A**) DLD-1-starved cells were incubated for different times with 100 μM P14, or for 30 min with 10% FBS. (**B**) DLD-1 starved cells were incubated for 3 h with 100 μM P14, P14A, P14B, P14C or P14D, or for 30 min with 10% FBS, or for 10 min with 50 ng/mL of EGF. (**C**) DLD-1 starved cells were incubated for different times with either 10% FBS or 100µM P14B. (**A**–**C**) The levels of activation and the total levels of RAF, MERK, ERK and AKT were analyzed by WB with specific antibodies against the active phosphorylated forms of these kinases or against the total forms of these proteins, respectively. In (**C**), tubulin was used as loading control. Tubulin 1 corresponds to P-AKT and P-MEK gel, and Tubulin 2 to P-ERK, P-RAF and RAF gel.

Since we hypothesized that the compounds binding to the pocket near the α5 helix might prevent the interaction of CaM with KRAS, and since we had previously shown that CaM inhibited KRAS signaling, we first analyzed the effect of P14 on the activation of downstream RAS signaling pathways (RAF/MEK/ERK and PI3K/AKT) in DLD-1 cells (CRC cells carrying one oncogenic KRAS-G13D allele). Although DLD-1 cells harbor a different oncogenic mutation in KRAS to the one of the structure we had used in Section 2.1 and Section 2.2, we know from our previous work that CaM binding to KRAS is not dependent on mutations in this region of the protein (both GTP-loaded KRAS-G12V and GTP-loaded wild type KRAS bind to CaM) [36,37]. DLD-1 serum-starved cells (0.1% FCS) were treated with P14 (100 µM) at different times, and the activation of AKT and ERK was evaluated by Western blot (WB). The data showed that P14 significantly increased P-ERK and P-AKT at 3 and 6 hours of cell treatment (Figure 2A). As a 3-h treatment was associated with major effects, we chose this duration for the subsequent experiments with the other compounds.

Next, DLD-1 serum-starved cells were treated with P14A-P14D at 100 µM for 3 h and activation of AKT and ERK was assessed as above. The results showed that P14, P14B, and P14C activated AKT and ERK kinases, with P14 and P14B being the most efficient, whereas P14D had no impact on cell signaling.

Since P14B was the compound with the greatest capacity to activate Ras signaling, even at levels comparable with those reached with 10% FBS (at 30 min), we selected it for further study. The kinetics of RAS downstream signaling was analyzed in P14B-treated cells compared to 10% FBS serum. As shown in Figure 2C, kinetics of AKT, MEK and ERK phosphorylation differed. ERK and MEK activation was clearly delayed in P14B-treated cells compared with 10% FBS-treated ones. Additionally, and especially in the case of ERK, its phosphorylation was maintained for a longer time. Ser338 phosphorylation of C-RAF was also induced but was not sustained for longer time. This suggested that some of the negative feedback pathways to deactivate MEK and ERK [11] were provably not induced by P14B, or alternatively that C-RAF activity was maintained even in the absence of Ser338 phosphorylation [44]. Thus, P14B—a small compound that was shown to interact with the α4-α5 surface of KRAS in silico—was able to induce a sustained increase in KRAS signaling.

### 2.4. P14B Binds Directly to Oncogenic KRAS and Competes with CaM

**Figure 3:**
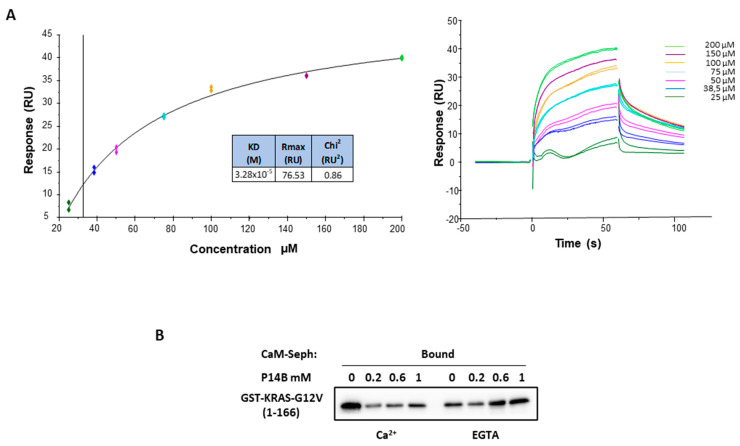
(**A**) Kinetic analysis (**left**) and sensograms (**right**) of the binding of P14B to GST-K-Ras-G12V (1–166) determined by surface plasmon resonance. RU, resonance units. The same code of colors indicated in the kinetic analysis are used in the sensogram to indicate each concentration of P14B (**B**) Competition between P14B and CaM for binding to oncogenic KRAS: CaM-sepharose pulldown assays were performed using GST-KRAS-G12V in the presence of P14B. Ca^+2^ or EGTA containing buffers indicate specific or non-specific KRAS-G12V binding to CaM, respectively. Bound fractions were analyzed by WB with anti-KRAS specific antibodies.

To confirm the direct interaction between KRAS and P14B, surface plasmon resonance analysis was performed. Purified GST-KRAS-G12V(amino acids 1 to 166) was immobilized and then GTP-loaded. After using different concentrations of P14B as the analyte, an affinity constant (KD) of 32.8 µM of P14B for GTP-loaded KRAS was determined (Figure 3A).

Given that P14B is able to bind to oncogenic KRAS (Figure 3), most likely via the same surface that KRAS uses to interact with CaM, we analyzed the possibility that P14B can displace CaM from oncogenic KRAS. To this end, we performed an in vitro competence assay that consisted of pulling-down recombinant GTP-loaded GST-KRAS-G12V (1-166) with CaM-sepharose beads followed by incubation with increasing concentrations of P14B. Figure 3B shows that Ca^+2^-dependent GST-KRAS-G12V binding to CaM was reduced in the presence of P14B.

### 2.5. P14B Favors the Interaction of Oncogenic KRAS with BRAF and Phosphorylated C-RAF

We assessed the possibility that P14B favors RAS-effector binding and consequently increases downstream RAS signaling. To this end, co-immunoprecipitation experiments were carried out to analyze the interaction of KRAS with different RAF family members upon P14B treatment. The interaction was analyzed after 10 min of P14B treatment since P-C-RAF and P-MEK were observed after 15 min (Figure 2C).

**Figure 4:**
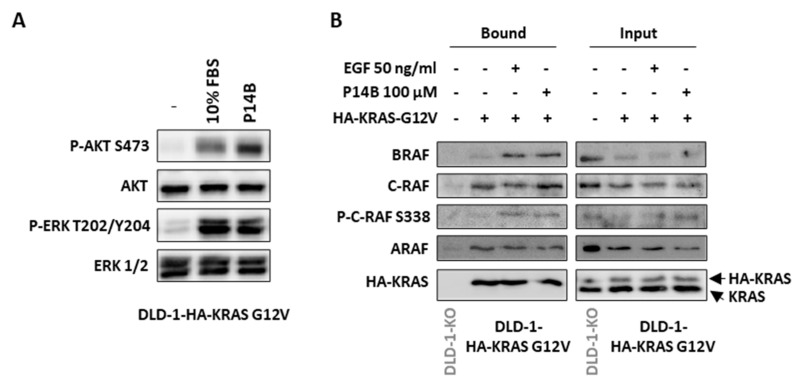
P14B treatment increases oncogenic KRAS interaction with its effectors BRAF and C-RAF/P-C-RAF. (**A**) Serum starved DLD-1 cells stably expressing HA-KRAS-G12V (DLD-1-HA-KRAS G12V) were incubated for 3 h with 100 μM P14B, or for 30 min with 10% FBS. WB was performed with the indicated antibodies. (**B**) Co-immunoprecipitation of HA-KRAS G12V with BRAF, C-RAF/P-C-RAF, or ARAF was analyzed in starved DLD-1-KO cells stably expressing HA-KRAS G12V after being treated with P14B (100 μM) or EGF for 10 min. IP was performed with anti-HA antibodies and WB of the bound and input fractions with anti-BRAF, anti-C-RAF, anti-P-C-RAF(S338), and anti-ARAF-specific antibodies. (-) non-treated.

DLD-1 cells stably expressing HA-KRAS-G12V were serum-starved and then treated for 10 min with either EGF or P14B. P14B induced phosphorylation of AKT and ERK in these cells (Figure 4A). This confirmed that the type of oncogenic mutation was not affecting P14B effects. As expected, the co-immunoprecipitation analysis showed that C-RAF interacted with oncogenic KRAS under serum-starvation conditions, but this immunoprecipitated C-RAF was phosphorylated only upon EGF treatment (Figure 4B). Interestingly, treatment with P14B increased the levels of P-C-RAF co-immunoprecipitated with KRAS to levels similar to those reached by EGF treatment (Figure 4B). In contrast, BRAF did not bind to oncogenic KRAS under serum-starved conditions and, upon EGF stimulation, the interaction of these two proteins was observed. Interestingly, P14B treatment induced the same effect as EGF and an increase in the KRAS–BRAF interaction was observed with respect to non-treated cells (Figure 4B). No differences were detected regarding KRAS–ARAF binding when cells were treated with P14B.

The assays presented here prove that the treatment of CRC cells with P14B intensifies oncogenic KRAS interaction with P-C-RAF (S338) and BRAF, in agreement with the positive impact of this compound on downstream RAS signaling.

### 2.6. Viability of CRC Cells Expressing Oncogenic KRAS Is Impaired by Treatment with P14B, but Not That of Normal Cells

**Figure 5:**
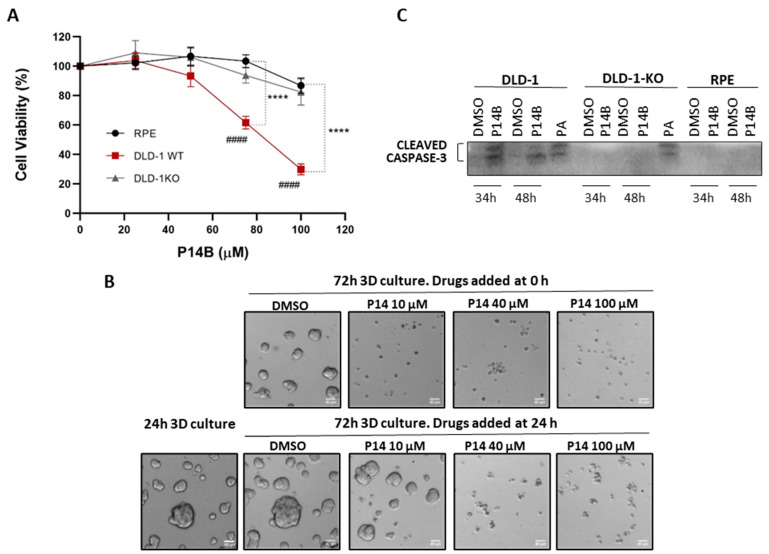
P14B decreases the viability of CRC cells expressing oncogenic KRAS. (**A**) Effect of P14B on the viability of DLD-1, DLD-1-KO and hTERT-RPE cells. Cells were treated with P14B at a dose ranging from 0 to 100 μM and incubated for 24 h, at which time cell viability was determined by MTS assay. The experiment was repeated at least three times. Differences were assessed using a one way ANOVA and Multiple Comparisons Test. **** means *p* < 0.0001 between DLD-1 and RPE or DLD-1-KO cells; #### means *p* < 0.0001 between different concentrations of P14B treatment and non-treated DLD-1 cells and considered significant when *p* ≤ 0.05. (**B**) 2.5 × 10^4^ DLD-1 cells were cultured on top of a thin basement membrane matrix (Matrigel) overlaid with a dilute solution of this matrix (3D on-top Matrigel assay). At the time of seeding or 24h later, cells were treated with P14B (10 μM, 40 μM or 100 μM), and 3 days after seeding the colonies formed were analyzed. Representative phase-contrast images are shown. All scale bars, 40 μm. (**C**) DLD-1 cells treated with P14B (100 μM) for 36 h and 48 h were lysed and the expression of the apoptosis marker cleaved caspase-3 was analyzed by WB. Cells treated with the apoptosis inducer palmitic acid (PA) were used as a positive control.

Since both the inhibition and sustained activation of RAS signaling are related to a decrease in cell survival, we analyzed the viability of the CRC cell lines DLD-1 (expressing oncogenic KRAS) and DLD-1-KO (oncogenic KRAS allele deleted), and the non-transformed cell line hTERT-RPE. Dose–response experiments (from 0 to 100 µM) at 24 h showed interesting results: P14B significantly reduced the cell viability of DLD-1, specifically at 75 µM and 100 µM. At the IC50 of approximately 80 µM for DLD-1 cells, the viability of DLD-1-KO and of non-transformed cells was not affected (Figure 5A). Interestingly, no significant differences in cell viability were observed between DLD-1-KO and hTERT-RPE.

The effect of P14B in DLD-1 cells cultured in 3D conditions was also determined. When P14B was added when seeding the cells, a drastic reduction in the capacity to form colonies in Matrigel was observed even at 10 µM. When P14B was added 24 h after seeding (when colonies were already formed), a clear reduction in the size of the colonies was observed at 40 µM (Figure 5B).

As it has previously been reported that sustained ERK signaling could induce an increase in autophagy [45], we analyzed the induction of autophagy in DLD-1 cells treated with P14B (Figure S5, Methods S2). To assess autophagic flux, cells were co-treated with P14B and concanamycin A (ConcA), a specific inhibitor of v-ATPase-dependent lysosome acidification. Our results showed that P14B did not significantly modify the autophagic flux, as no differences were observed in the autophagic markers protein levels, LC3-II and SQSTM1, upon treatment with P14B for 6 or 12 h (Figure S5). Similarly, P14B did not increase autophagic vesicles’ size or number (Figure S5), suggesting that P14B function is independent of autophagy in DLD-1 cells.

Finally, to determine whether cells were dying by apoptosis, the three cell lines were treated with P14B for 34 h or 48 h, and the protein expression of cleaved caspase-3 was analyzed by WB. As shown in Figure 5C, cleaved caspase-3 was only detected in DLD-1-treated cells being undetectable in the samples of DLD-1-KO and hTERT-RPE.

Consequently, we conclude that P14B selectively reduces the viability of CRC cells expressing oncogenic KRAS, causing minor effects if these cells have been knocked out for oncogenic KRAS or in non-transformed cells. Thereby, P14B might be considered as an inducer of apoptosis in CRC with oncogenic KRAS.

### 2.7. Comparison of Ras Signaling between DLD-1, DLD-1-KO and hTERT-RPE Cells Treated with P14B

Since important differences were observed between the viability of cells treated with P14B depending on their expression or lack of expression of oncogenic KRAS, we further studied whether these discrepancies were also reflected in KRAS downstream signaling.

**Figure 6:**
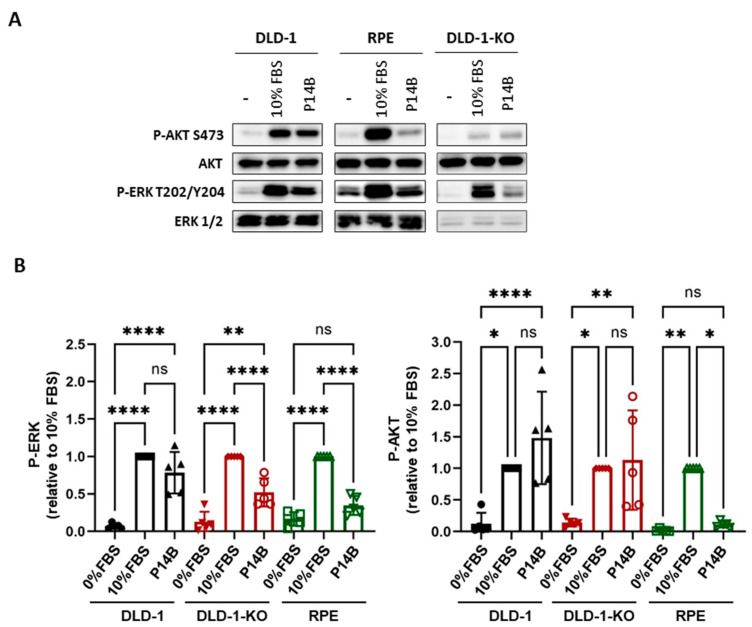
P14B treatment of CRC cells expressing oncogenic KRAS increases endogenous downstream RAS signaling. (**A**) DLD-1, DLD-1-KO and hTERT-RPE starved cells were incubated with 100 μM of P14B for 3 h and the levels of activation of ERK and AKT were analyzed by WB. Specific antibodies against the active phosphorylated and total proteins were used. (**B**) Graph showing quantification of four independent experiments. Differences were assessed using a one-way ANOVA and Multiple Comparisons Test, and considered significant when *p* ≤ 0.05. *: *p*-value < 0.05; **: *p*-value < 0.01; ****: *p*-value < 0.0001; and ns: non-significant.

Serum-starved cells (0.1% FCS) were treated with P14B (100 µM) and the activation of AKT and ERK was evaluated by WB. Results show that while AKT and ERK phosphorylation dramatically increased after the P14B treatment of DLD-1 cells, reaching levels comparable with those reached with 10% FBS, no significant increase was observed in hTERT-RPE cells treated with P14B. Regarding DLD-1-KO, AKT and ERK activation by P14B was also observed, but the levels of ERK phosphorylation were significantly lower than the ones reached upon 10% FBS treatment (Figure 6). Thus, cell death observed in the different cell lines positively correlated with an increase in ERK activation.

## 3. Discussion

Using molecular modeling techniques, we searched for a druggable pocket in the α4-α5 helices regions of KRAS, which we previously described as being important for the interaction with CaM [36,37]. Docking and molecular dynamics technologies allowed us to identify a compound that can be accommodated in this pocket and which the experimental data confirmed interacts with KRAS, inhibits CaM binding and modulates Ras signaling.

The α4-α5 helices surface of KRAS allows its interaction with non-effector proteins, reinforces the binding of some effector proteins [26], facilitates KRAS clustering and dimerization [20,22,46], and modulates KRAS orientation with respect to the plasma membrane [47]. Since all these events ultimately modulate the activity of oncogenic KRAS, the identification of compounds that can bind to this surface and disrupt these protein–protein interactions has gained interest in recent years. In fact, Gorfe’s group previously described a pocket (which they named P3) that corresponds to the one we describe here, and identified compounds that bind to it and that reduced the levels of wild-type GTP-RAS in glioblastoma cells [16]. Additionally, a monobody (NS1) that interacts with the α4-β6-α5 helix surface of HRAS and KRAS was also generated. NS1 inhibited dimerization and clustering of these RAS isoforms and hence attenuated oncogenic HRAS and KRAS signaling [29]. Interestingly, this molecule inhibited cell transformation induced by oncogenic KRAS and has antitumoral properties [35]. Using computational analyses and a combination of computational and biochemical approaches, Stockwell’s group identified a compound (KAL-21404358) that binds to the same pocket region of KRAS, which they named the P110 site (because it is near proline 110). This compound impaired the interaction of KRAS-G12D with BRAF and disrupted the RAF-MEK-ERK and PI3K-AKT signaling pathways [33]. In contrast to these findings, the compounds that we identified are activators of RAS signaling instead of inhibitors. Although they all interact with the same KRAS surface, the specific amino acids of KRAS interacting with the compounds may differ and, hence, the differences observed with KRAS signaling. For instance, in contrast to KAL-21404358, our two compounds, P14 and P14B, interact with Glu168 and Lys169 of KRAS.

Since our aim was to analyze the effect on signaling pathways activated by oncogenic KRAS, we used DLD-1 cells, which are CRC cancer cells harboring one oncogenic KRAS allele. Even in the presence of this oncogenic allele, the addition of FBS to serum-starved cells induced an increase in P-MEK, P-ERK, and P-AKT levels. That means that additional signals induced by growth factors allow full activation of the signal transduction pathways. Remarkably, the addition of P14B alone was also enough to achieve this activation. Furthermore, ERK phosphorylation was more sustained in P14B-treated cells than in FBS-treated cells. This suggested that some of the negative feedback pathways to deactivate ERK [11] were provably not induced by P14B. In agreement with the induction of MEK and ERK phosphorylation in cells treated with P14B, we also observed changes in RAF binding to RAS. As expected, oncogenic KRAS was able to bind to C-RAF even in serum-starved conditions, but growth factor stimulation was necessary to allow phosphorylation and hence activation of this C-RAF bound to KRAS [48]. In contrast, BRAF binding to oncogenic KRAS was not observed in serum-starved DLD-1 cells. Interestingly, our data show that P14B treatment completely mimicked the effect of growth factors regarding the presence of P-C-RAF and BRAF bound to oncogenic KRAS.

As intended, in this study we determined how P14B interacts with the globular region of oncogenic KRAS and showed that it inhibits the interaction of CaM with this domain of KRAS. Our previous studies indicated that CaM interacts with KRAS in vivo and that inhibition of CaM enhances both wild-type and oncogenic KRAS signaling. This could explain the increased KRAS downstream signaling observed in P14B-treated cells. The disengagement of CaM interaction with the α4-α5 surface could facilitate interaction with effectors, such as RAF, within this region. In fact, the CRD (cysteine rich domain) of RAF is required for full RAF binding to RAS and the interactions are mediated via residues in the inter-switch region and helix α5 (R149, D153, and Y157) [9,26]. Alternatively, since CaM inhibits KRAS phosphorylation at Ser181 [38], P14B could indirectly favor KRAS phosphorylation by preventing CaM interacting with KRAS. Accordingly, we have previously shown that oncogenic KRAS with a mutation mimicking this phosphorylation (Ser181D) segregates in specific nanoclusters and has increased affinity with P-C-RAF and P110 PI3K [38]. Thus, by facilitating KRAS phosphorylation, P14B could segregate oncogenic KRAS into specific nanoclusters, where effectors are more accessible, and C-RAF phosphorylation is facilitated.

Moreover, P14B could directly modulate the interaction of other proteins with the α4-α5 helices region of KRAS independently of preventing the CaM interaction. For instance, in the context of ovarian, lung and pancreatic cancer, the tumor suppressor DIRAS3 binds to the α5 helix region of KRAS in the same pocket as P14B [25]. DIRAS3 prevents KRAS clustering and inhibits its signaling. It would be interesting to determine whether DIRAS3 has a similar role in CRC cells and whether P14B inhibits this interaction. On the other hand, it has been proposed that RAS proteins assemble into higher-order helical complexes, named Ras-Raf signalosomes, where not all RAS members directly contact the plasma membrane and the β4–α3, β5–α4 and β6–α5 loops of KRAS are involved in the formation of asymmetric KRAS dimers [21]. It would be interesting to determine whether P14B enhances the generation of this superstructure.

Remarkably, we have shown that P14B increased apoptosis in DLD-1 cells harboring an oncogenic KRAS allele. This is apparently contradictory but the promotion of apoptosis by RAS, and specifically the RAS/RAF/ERK pathway, has been described previously [49,50]. As mentioned above, ERK phosphorylation was more sustained in P14B-treated cells than in FBS-treated cells and there is evidence that a sustained ERK activation induces a decrease in cell survival [11,51]. In agreement with that evidence, DLD-1 cells knock-out for the oncogenic allele of KRAS and non-transformed hTERT-RPE do not show such a high increase in ERK phosphorylation upon P14B treatment and did not undergo cell death upon P14B treatment. It is also possible that additional unknown KRAS effectors are engaged by P14B and not by FBS treatment and that those effectors are the ones responsible for the observed cell death. Consistent with our results, another small drug agonist of KRAS (which binds the GTP/GDP-binding pocket of KRAS and leads to the accumulation of GTP-KRAS) was previously reported to induce the apoptotic and autophagic cell death of lung cancer cells [52].

In conclusion, our results corroborate the hypothesis that the α4-α5 helices region of KRAS is relevant for KRAS signaling, and that drugs designed to interact with this site can induce the enhanced signaling of oncogenic KRAS that leads CRC to cell death.

## 4. Materials and Methods

### 4.1. KRAS Modelling

#### 4.1.1. Pocket and Structure Selection

Eight KRAS structures with PDB codes 4EPR, 4EPY, 4EPT, 4EPV, 4EPW, 4EPX, 4DST, and 4DSN were used to search and identify possible allosteric binding sites of KRAS. To this end, ligands and other molecules were removed, when present, and the Site Finder feature of MOE was used.

#### 4.1.2. Preparation of K-Ras4B System

The initial structure, with PDB code 4DSN, was prepared by removing molecules other than the cofactor and changing the GCP residue to GTP. Then, the protein was protonated using the Protonate function of MOE fixing a pH = 7. The parameters to describe the GTP cofactor were obtained with the antechamber [53] module of AMBER14 [54]. However, the phosphate parameters were replaced with those of the Bryce group (http://www.pharmacy.manchester.ac.uk/bryce/amber, accessed on 5 October 2022). Missing residues in the HVR region (from 178 to 188) were added. The ff99SB [55] and the GAFF [56] force fields were used for the protein and the cofactor respectively. The AM1-BCC charges [57] for the GTP were used. Next, the complex was placed in a cubic periodic box filled with TIP3P water molecules [57], imposing a minimal distance of 15 Å between the protein and the box walls. Water molecules closer than 2.2 Å to any protein atom were removed and neutralizing counter-ions were added at positions of lowest electrostatic potential.

#### 4.1.3. Energy Minimization

All the simulations were carried out using the pmemd program of the AMBER14 Software [54] on a graphics processing unit (GPU). Before starting the molecular dynamics calculation, the structure was energy minimized in a multistep procedure. First, the minimization of the water molecules and counter-ions was performed through 5000 steps of the conjugate gradient algorithm keeping the rest of the system fixed with a constant force of 10 kcal/mol Å. Second, the minimization of the side chains of the complex was performed in four stages using a decreasing force constant of 10, 5, 1 and 0,5 kcal/mol Å to restrain the backbone atoms. Finally, 10,000 steps of the conjugate gradient method were carried without restrictions out to minimize the whole system.

#### 4.1.4. K-Ras cMD Simulations and Trajectory Analysis

The minimized system was used as a starting point for the cMD simulations. Molecular dynamics was performed at 300K, so the system was first heated up in a step wise manner at a rate of 30 K every 20 ps. The protein backbone atoms were restrained with a force constant of 0.5 kcal/mol·Å. After the system was at the right temperature, we per-formed 200 ps of simulation at constant pressure (NPT ensemble) without any restraint in order to allow density equilibration. Finally, a cMD simulation of 50ns/80ns length was performed. The temperature was regulated by using the Berendsen algorithm with a time coupling constant of 0.2 ps. All bonds involving hydrogen atoms were constrained to their equilibrium value using the SHAKE algorithm, allowing the use of a 2 fs integration time step in all of the simulations. Non-bonded interactions were truncated at a cut-off of 10 Å, and long-range electrostatic interactions were treated with the particle-mesh Ewald method (PME).

To analyze the trajectories once the simulations were performed, the AMBER cpptraj module was used [58].

#### 4.1.5. Binding Energy Calculations

The Molecular Mechanics Poisson Boltzmann (Generalized Born) Solvation Area MM/PB(GB)SA was used as an implementer in the MMPBSA module of AMBER to calculate the free energy change between two states [59]. In this method, the free binding energy of the system is obtained as the sum of its gas-phase energy, the solvation free energy and a contribution due to the entropic change. The solvation free energy is obtained by summing the polar and non-polar contributions. The polar contribution can be calculated either with the Poisson-Boltzmann (PB) equation or with the Generalized Born (GB) model. The non-polar contribution is estimated by a term proportional to the solvent accessible sur-face area (SASA). For PB calculations we used the by omission parameters and for the GB calculations we used option 5 of the MMPBSA.py module [60].

### 4.2. KRAS Docking

Before starting the proper docking, K-Ras selected structures were extracted from the trajectory of the MD simulation and water molecules and counter-ions were removed. The docking process was performed with MOE. Two different databases were studied. For conformational analysis, the set of ligands was treated in a flexible manner by rotating rotatable bonds. Since all the analyzed molecules were rather small, the systematic meth-od was used to search for all the possible structures, setting a conformational limit of 300 structures. In a first step, receptor structures remained fixed. For placement, we used the Tri-angle Matcher method, and the Affinity dG scoring function was used to assess candidate poses. After performing the docking of all the compounds of the database, those compounds with a binding energy higher than zero were removed. In a second step, a refined docking with induced fit—which allows the free movement of the ligand inside the pocket as well as the movement of the lateral chain of the nearby residues to accommodate the ligand—was performed with the 1000 best molecules of the first step.

#### Binding Analysis of the Selected Compounds

The specificities of the binding of the selected compounds were analyzed by per-forming cMD simulations of K-Ras with either ligand. The same methodology as before was followed to prepare the system and carry out the minimizations. Afterwards, the binding free energy of the protein with the ligand during the time was analyzed with the MM/PB(GB)SA methodology.

### 4.3. Synthesis of P14 Derivatives

Described in Supplementary Methods S1 section.

### 4.4. Cell Lines and Culture Conditions

DLD-1 (KRAS^WT/G13D^) (clone V15, #HD PAR-086) colorectal adenocarcinoma cell line and DLD-1 knockout of mutant KRAS allele, DLD-1 KO (KRAS^WT/-^) (clone DWT7, #HD105-002), were obtained from Horizon Discovery Ltd. (Cambridge, UK). hTERT-RPE (KRAS^WT/WT^) immortalized retinal pigment epithelial human cell line was obtained from the American Tissue and Cell Collection (ATCC). DLD-1 KO cells stably expressing HA-KRAS G12V were previously generated in our laboratory [61]. All cells were grown in DMEM-HAM’s F12 (1:1) supplemented with 10% fetal bovine serum (Biological Industries, HAEMEK, Israel), penicillin, streptomycin, and nonessential amino acids. Cells were tested once per month for mycoplasma contamination.

### 4.5. Drug Treatment and EGF-Dependent Signalling Activation

Cells were seeded in a media containing 10% FBS for 24 h and then they were serum starved for the next 24 h. Afterwards, they were incubated with the different compounds during the times specified in each figure. When indicated, also in order to activate downstream KRAS cell signaling, treatment for 10 min with EGF (50 ng/mL) (Sigma-Aldrich St Louis, MO, USA) was performed.

### 4.6. Western Blot (WB) and Antibodies

Proteins were resolved by SDS-PAGE and transferred onto PVDF membranes (Immobilon-P, Millipore, Burlington, MA, USA), and Western blot was performed as previously described [61]. The following primary antibodies were used: anti- c-RAF (610151 BD Transduction, Franklin Lakes, NJ, USA; 1:500); anti-phospho-c-RAF S338 (9427 Cell Signaling; 1:500); anti-AKT (Cell Signaling 9272, 1:1000); anti-phospho-AKT T308 (4056 Cell Signaling, Danvers, MA, USA; 1:1000); anti-phospho-MEK1/2 S221 (2338 Cell SignalingDanvers, MA, USA; 1:000); anti-p44/42 MAPK (ERK1/2) (9102 Cell Signaling, Danvers, MA, USA; 1:2000); anti-phospho-p44/42 MAPK(ERK1/2) T202/Y204 (4370 Cell Signaling, Danvers, MA, USA; 1:2000); anti-HA (H6908 Sigma-Aldrich, St Louis, MO, USA; 1:1000); anti-cleaved-caspase-3 (Asp175) (9661 Cell Signaling, Danvers, MA, USA; 1:1000); anti-CDK4 (sc-749 Santa Cruz, Dallas, TX, USA; 1:500); anti-LC3 (PM036 MBL, Tokyo, Japan; 1:1000); anti-p62/SQSTM1 (M162-3 MBL, Tokyo, Japan; 1:1000); anti-β-actin (A2228 Sigma-Aldrich, St Louis, MO, USA, 1:1000). HRP-coupled secondary antibodies used: goat anti-rabbit (170-6515 BioRad, Hercules, CA, USA; 1:3000) or goat anti-mouse (170–6516 BioRad, Hercules, CA, USA; 1:3000).

For analysis of RAS signaling, cells were lysed in a buffer containing 67 mM Tris-HCl pH 6.8 and 2% SDS and then the samples were heated at 97 °C for 15 min. Protein concentration of the lysates was assessed using the Lowry method. An aliquot of 15 µg of protein per sample was loaded onto the gels. 

### 4.7. Immunofluorescence

Described in Supplementary Methods S2 section.

### 4.8. Surface Plasmon Resonance Analysis

Surface Plasmon Resonance Analysis was performed by using BIAcore T200 equipment. GST-KRAS-G12V (1-166) and GST were covalently immobilized on two of the channels of CM5 Series S Chip following the manufacturer’s instructions. KRAS was loaded with GTP by injecting 1 mM of GTP 10 µL/min for 30 min in exchange buffer (20 mM Tris-HCl pH 7.5; 50 mM NaCl, 5% glycerol, 0.1% Triton X-100, 1 mM DTT) with 10 mM EDTA at 30 °C, and loading was blocked by injecting exchange buffer with 15 mM MgCl2 at 10 μL/min for 5 min. P14B was injected at 12,5 µM, 25 µM, 38.5 µM, 50 µM, 75 µM, 100 µM and 150 µM in running buffer (150 mM NaCl, 50 mM Tris-HCl pH7.5, 2 mM MgCl2, 0.1% Triton and 5% DMSO), at a flow rate of 60 μL/min for 1 min, at 25 °C. Dissociation was allowed for 10 min in the same buffer. All runs were performed in duplicate. Nonspecific binding was subtracted by using two linked channels (GST-KRAS-G12V minus GST). Diverse solutions (from 3% to 8%) with DMSO were also prepared to analyze its effect on the RUs and a solvent correction was performed to reduce the error associated with the injection of the sample.

### 4.9. Co-Immunoprecipitation

DLD-1 KO cells stably expressing HA-KRAS-G12V were serum starved for the next 24 h before treatment with the compounds for the times indicated. Next, an IP with anti-HA antibody crosslinked to agarose beads (clone HA-7, A20956 Sigma-Aldrich; St Louis, MO, USA) was performed as previously described [61].

### 4.10. Calmodulin (CaM)-Pull Down

Then, 5 µg of recombinant GST-KRAS-G12V (1-166) (GTP loaded) and 12.5 µL of CaM-sepharose beads (Cytiva; Merck, Darmstadt, Germany) (previously washed with pull-down buffer (PDB) containing 50 mM Tris-HCl, pH 7.5, 150 mM NaCl, 0.1% (*v*/*v*) Triton X-100) was incubated in the presence of 1 mM CaCl_2_ or 5 mM EGTA (in a total volume of 100 µL with PDB) for 60 min at room temperature. The unbound fraction was collected by centrifugation and the bound fraction was washed four times with PDB with either CaCl_2_ or EGTA. The entire bound fraction was analyzed by WB.

### 4.11. Purification of GST-KRAS-G12V (1-166)

GST-KRAS-G12V (1-166) fusion protein was expressed in *Escherichia coli* BCl21 and then purified and loaded with the specific nucleotide as previously described [39].

### 4.12. Cell Viability Assay

Next, 10,000 cells in 50 µL of 10% FBS-containing medium were cultured for 24 h and then treated with the compounds (50 µL final volume) for 48 h hours in each well of a 96-well plate (100 µL final volume). An MTS viability assay (CellTiter 96^®^ Aqueous One Solution Cell Proliferation Assay, G3580, Promega, Madison, WI, USA) was assessed following the company conditions. The absorbance of each well was measured with a multimode plate reader (Spark, Tecan) at 490 nm. The percentage of cell viability was calculated by dividing the absorbance of each well by the average absorbance of the control wells (control wells had no significant differences among them when Students’ T-Test was applied). 

### 4.13. 3-Dimensional (3D) Cell Culture

A 3D on-top Matrigel assay was performed as described in [61].

### 4.14. Statistical Analysis

Statistical analyses were performed with GraphPad Prism 8.1. Data shown represent the mean ± SEM or SD (as indicated in figure legends) of three or four independent experiments. Differences were assessed using one-way ANOVA with multiple comparisons tests and were considered significant when *p* < 0.05.

## 5. Patents

The compounds presented here have been protected.

## Data Availability

Data is contained within the article or supplementary material.

## Supplementary Materials

The following supporting information can be downloaded at: https://www.mdpi.com/article/10.3390/ijms24010748/s1. Supplementary figures file: Figure S1. Evolution of ΔG_binding_ versus time for the K-Ras4B simulations using the MMGBSA (in orange) and MMPBSA (in blue) algorithms. Figure S2. Evolution of ΔG_binding_ versus time for the KRAS/P14 complex simulations using the MMGBSA (in orange) and MMPBSA (in blue) algorithms. Figure S3. Evolution of ΔG_binding_ versus time for the KRAS/P14B complex simulations using the MMGBSA (in orange) and MMPBSA (in blue) algorithms. Figure S4: P14 derivatives. Figure S5: P14B function is independent of autophagy in DLD-1 cells. Supplementary methods file: Methods S1: Synthesis of P14 derivatives. Methods S2: Immunofluorescence. Supporting information file: Original images for blots.
