# Supplementary material for: α4-α5 Helices on Surface of KRAS Can Accommodate Small Compounds That Increase KRAS Signaling While Inducing CRC Cell Death
Source: Int J Mol Sci. 2023 Jan 1;24(1):748. doi: 10.3390/ijms24010748 (PMC9821572; doi:10.3390/ijms24010748)
Supplement: Supplementary file 1 [file ijms-24-00748-s001.zip › SUPPLEM METHODS_Abuasaker et.al_051022.pdf]

SUPPLEMENTARY METHODS:

Methods S1: Synthesis of P14 derivatives

The preparation of the analogous compounds of **P14** was carried out under the conditions of a multicomponent reaction from hydrazine hydrate, methyl acetoacetate and the corresponding aldehyde or ketone (Scheme 1).

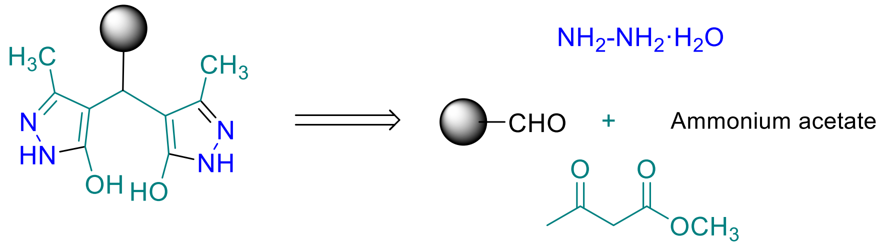

Scheme 1. Retrosynthesis of **P14** derivatives

It is a Knoevenagel-type condensation reaction between the aldehyde or ketone and the keto-ester and double addition of the hydrazine to form the pyrazole rings (Scheme 2). It is important to control the concentration as well as the p*H* of the solutions used in the extraction of the product and in the purification, to avoid as much as possible the formation of tautomeric systems.

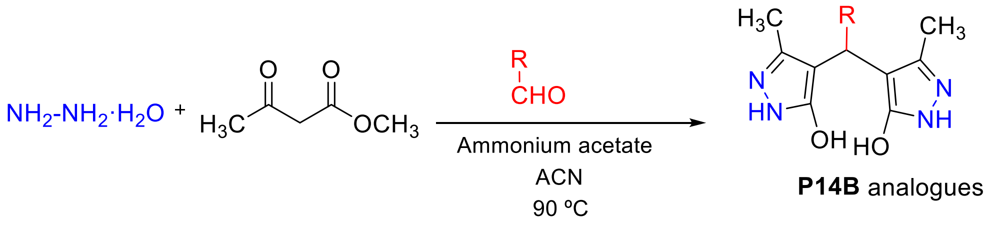

Scheme 2. Preparation of **P14B** analogues

The preparation of the aldehydes or ketones were carried out by means of cross-coupling reactions between substituted aryl halides and the relevant phenols or anilines catalyzed by Pd-complexes (Buschwald-Hartwig methode) (Scheme 3). For this, the classic organic synthesis methodology was followed or new conditions were developed.

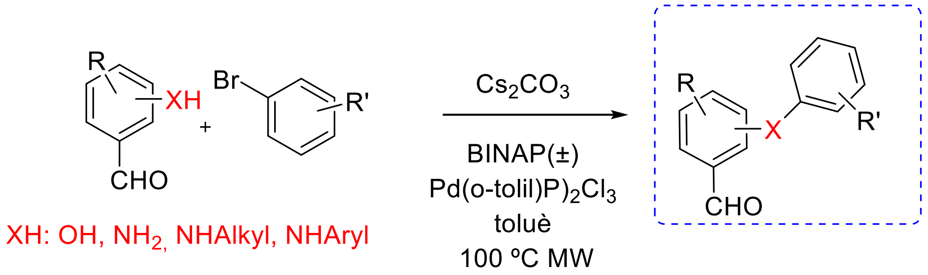

Scheme 3. Preparation of intermediates **aldehydes or ketones**

It should be noted that techniques such as the use of microwaves (MW) and automated purification were used for chemical synthesis to establish methodologies that are more

compatible with the environment. These techniques allow working with less solvents (from 60 to 80% less than using the classic methods) and also reduce the reaction time.

All the prepared compounds were analyzed by NMR techniques (^{1}H-NMR and ^{13}C-NMR) and the structure was confirmed. The purity of the compounds was determined by elemental analysis (C, H, N) and also by mass spectrometry of high resolution (HMRS, ESI).

Methods S2: Immunofluorescence

DLD-1 cells were grown as monolayers on coverslips and were treated for 12 h with P14B (100 μM) in the presence or not of concanamycin A (10 nM). Cells were fixed with 4% paraformaldehyde (PFA) for 10 min at room temperature. After 5 min washing with PBS, cells were permeabilized using 0.1% Triton X-100 in PBS for 10 min at room temperature. Then, cells were blocked with 10 mM glycine (Sigma-Aldrich, G8898) 1% BSA 0.01% Triton X-100 in PBS for 30 min at room temperature. Cells were incubated with primary antibodies [anti-LC3 (MBL PM036, 1:100) and anti-LAMP2 (BD Biosciences, 1:300) for one hour at room temperature. After several washed with PBS, cells were incubated with secondary antibodies for 45 min at room temperature [anti-mouse Alexa Fluor 488 (Invitrogen A11001, 1:400) and anti-rabbit Rhodamine Red-X (Invitrogen R6394, 1:400). Coverslips were then mounted using Vectashield Mounting Solution containing DAPI (Vector Laboratories, H-1200). Images were acquired with Carl Zeiss LSM880 confocal microscope and Zeiss ZEN software (Oberkochen, Germany). Fluorophores were excited with Argon 488, DPSS 561 and Diode 405 lasers. Image analysis was performed using FIJI ImageJ software (NIH, USA).
