# Supplementary material for: α4-α5 Helices on Surface of KRAS Can Accommodate Small Compounds That Increase KRAS Signaling While Inducing CRC Cell Death
Source: Int J Mol Sci. 2023 Jan 1;24(1):748. doi: 10.3390/ijms24010748 (PMC9821572; doi:10.3390/ijms24010748)
Supplement: Supplementary file 1 [file ijms-24-00748-s001.zip › SUPPLEM FIGURES_Abuasaker et.al_051022.pdf]

SUPPLEMENTARY FIGURES:

Figure S1:

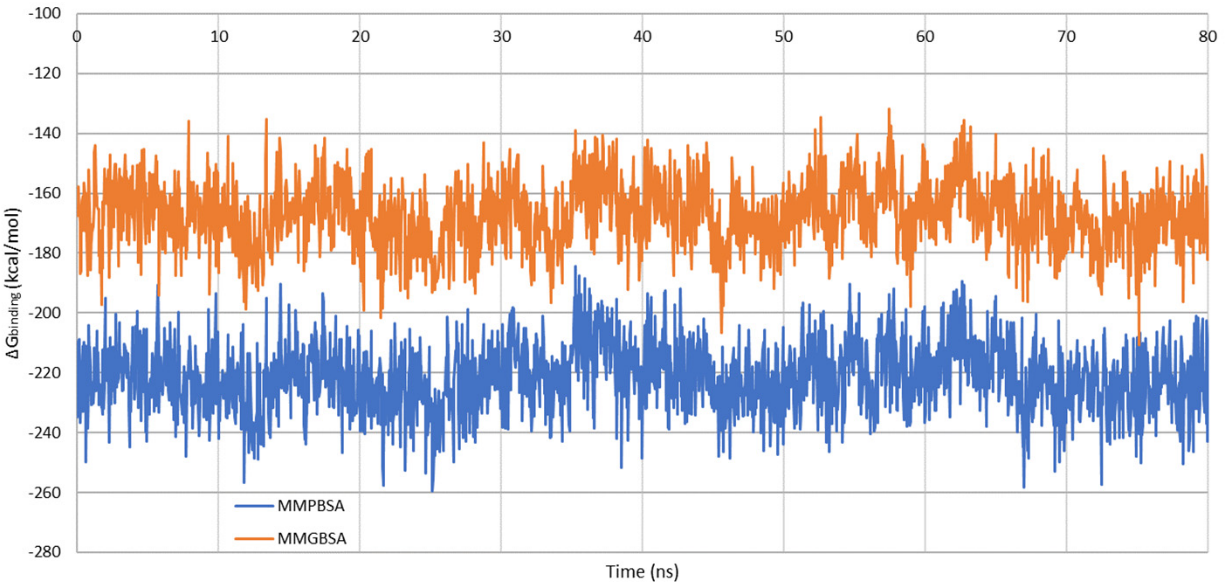

Figure S1. Evolution of $\Delta G_{binding}$ versus time for the K-Ras4B simulations using the MMGBSA (in orange) and MMPBSA (in blue) algorithms.

Figure S2:

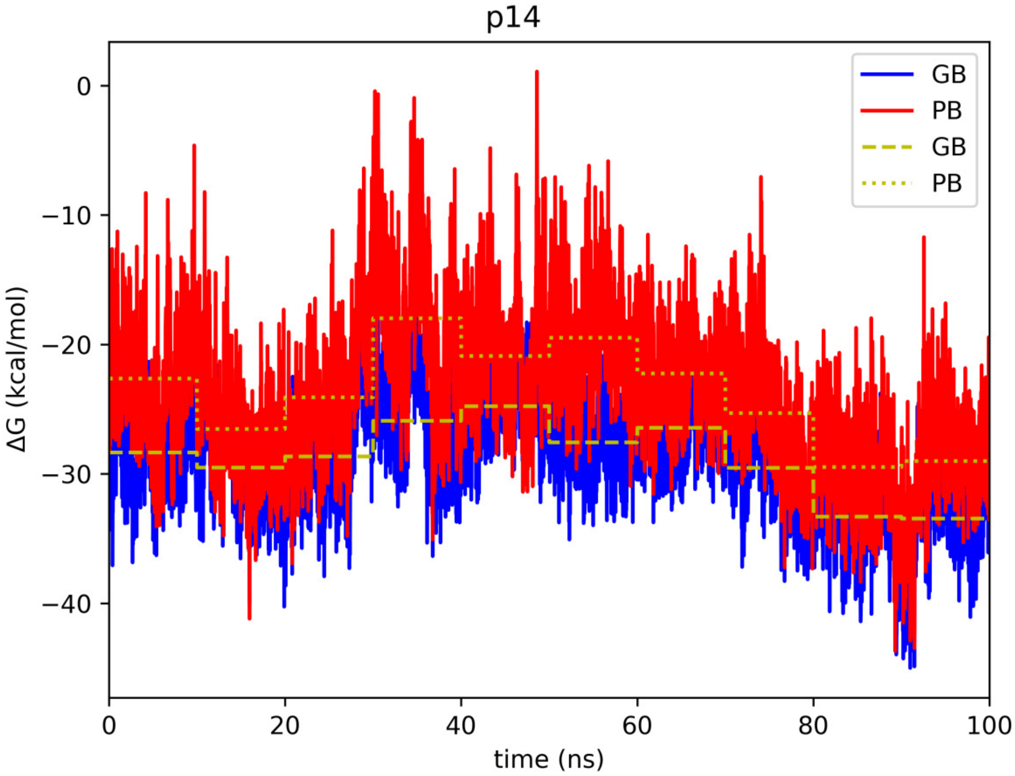

Figure S2. Evolution of ΔGbinding versus time for the KRAS/p14 complex simulations using the MMGBSA (in orange) and MMPBSA (in blue) algorithms.

Figure S3:

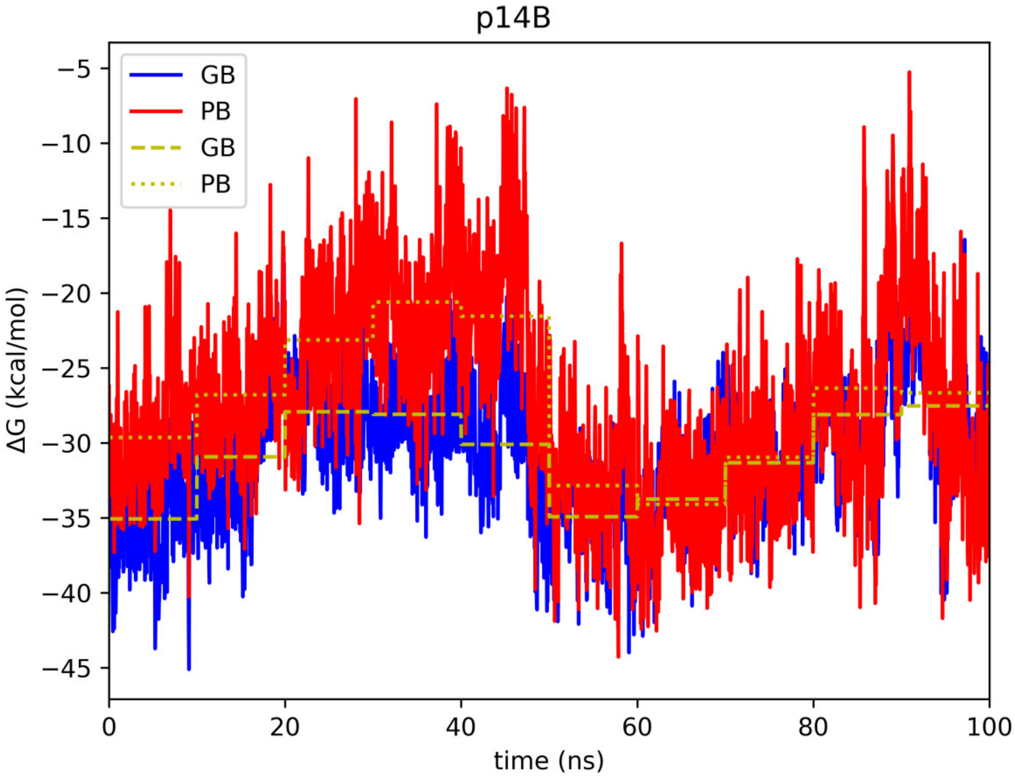

Figure S3. Evolution of $\Delta G_{binding}$ versus time for the KRAS/p14B complex simulations using the MMGBSA (in orange) and MMPBSA (in blue) algorithms.

Figure S4:

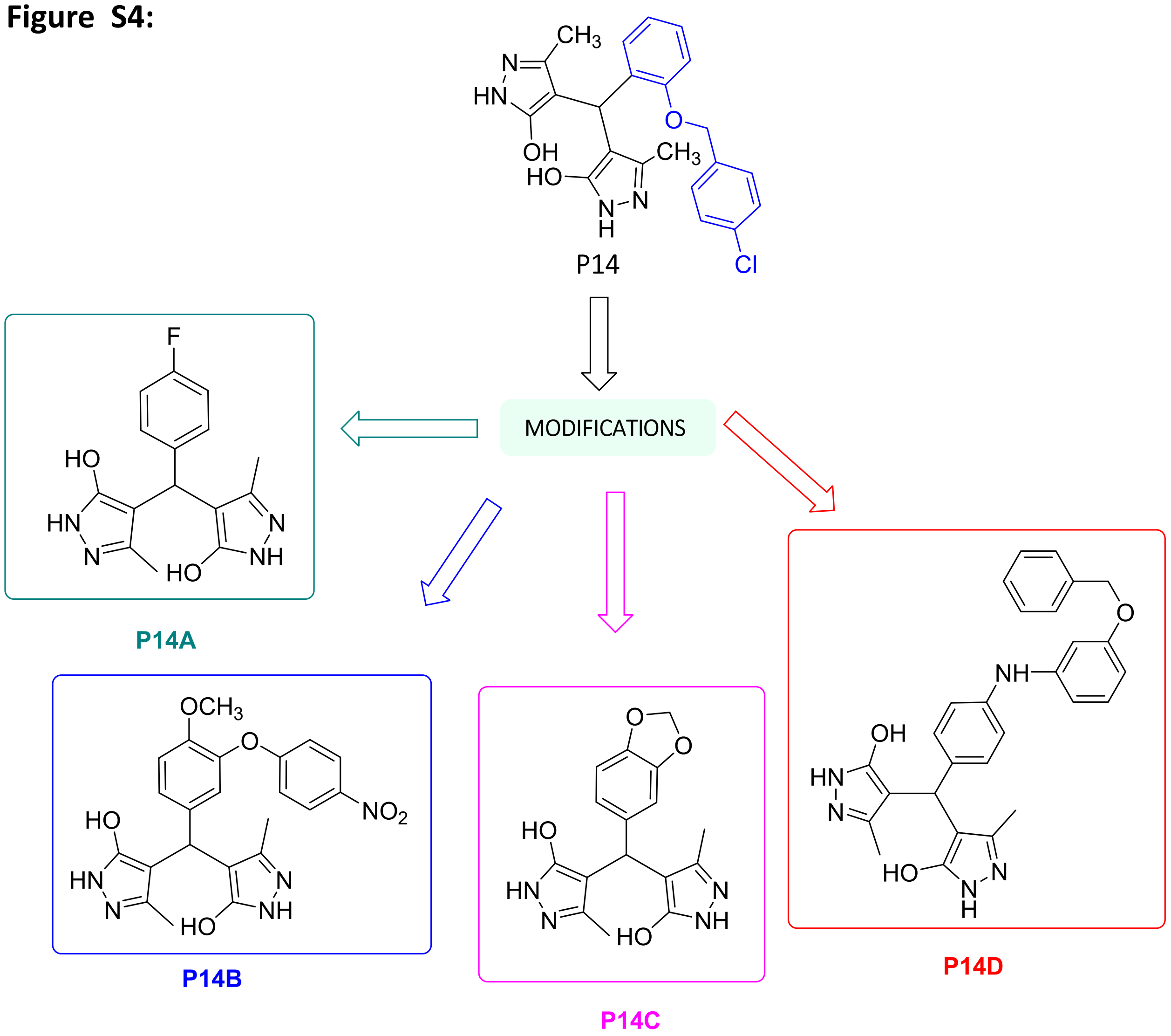

Figure S4. P14 derivatives

Figure S5:

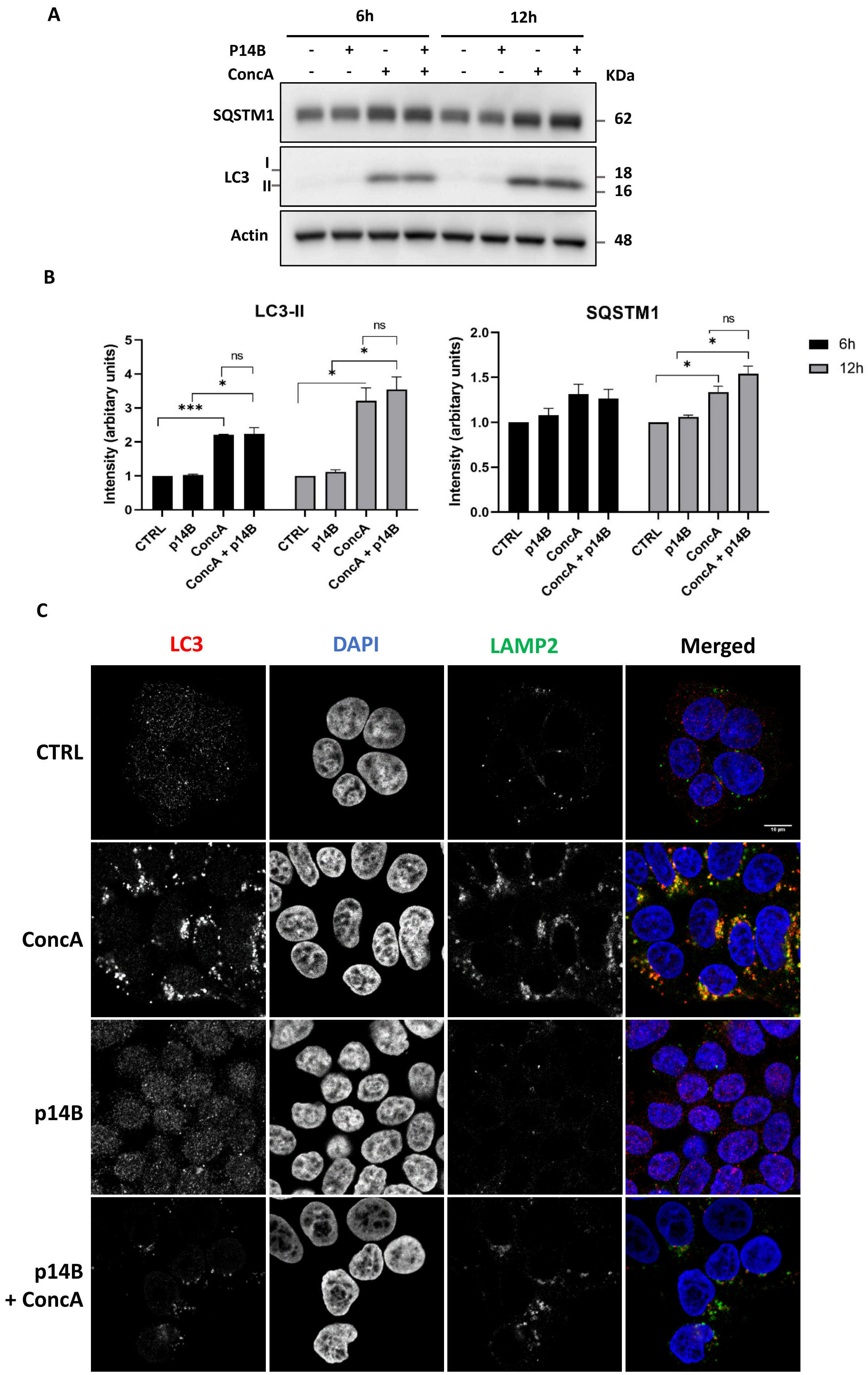
A
6h
12h
P14B
ConcA
KDa
SQSTM1
62
LC3
I
II
18
16
Actin
48
B
LC3-II
Intensity (arbitary units)
ns
*
***
CTRL
p14B
ConcA
ConcA + p14B
SQSTM1
6h
12h
C
LC3
DAPI
LAMP2
Merged
CTRL
ConcA
p14B
p14B + ConcA

Figure S5. **A**. **P14B function is independent of autophagy in DLD-1 cells.** DLD1 cells were treated with P14B (100 mM), Concanamycin A (10 nM) alone or combined for 6 and 12 hours. LC3 and SQSTM1 (p62) protein levels were detected by specific antibodies by western-blot and used as a readout for the analysis of the autophagic flux under the experimental conditions. b-actin protein levels served as loading control.

B. Quantification of LC3-II (left) and SQSTM1 (right) protein levels normalized to b-actin. Statistical significance is represented as: * $p < 0.05$, ** $p < 0.005$, *** $p < 0.001$, ns: non-significant. **C**. Representative confocal images of DLD1 cells treated as in panel A for 12 hours. Immunofluorescence was performed using specific antibodies against the autophagic marker LC3 (red) and lysosomal protein LAMP2 (green). Cells were counterstained with DAPI for nuclear detection (blue). Scale bar, 10 mm.
